# Alterations in the social-conditioned place preference and density of dopaminergic neurons in the ventral tegmental area in Clsnt2-KO mice

**DOI:** 10.18699/VJGB-23-14

**Published:** 2023-04

**Authors:** I.N. Rozhkova, S.V. Okotrub, E.Yu. Brusentsev, K.E. Uldanova, E.А. Chuyko, V.A. Naprimerov, T.V. Lipina, T.G. Amstislavskaya, S.Ya. Amstislavsky

**Affiliations:** Institute of Cytology and Genetics of the Siberian Branch of the Russian Academy of Sciences, Novosibirsk, Russia; Institute of Cytology and Genetics of the Siberian Branch of the Russian Academy of Sciences, Novosibirsk, Russia; Institute of Cytology and Genetics of the Siberian Branch of the Russian Academy of Sciences, Novosibirsk, Russia; Institute of Cytology and Genetics of the Siberian Branch of the Russian Academy of Sciences, Novosibirsk, Russia; Institute of Cytology and Genetics of the Siberian Branch of the Russian Academy of Sciences, Novosibirsk, Russia; Institute of Cytology and Genetics of the Siberian Branch of the Russian Academy of Sciences, Novosibirsk, Russia Novosibirsk State Agricultural University, Novosibirsk, Russia; University of Toronto, Toronto, Canada; Scientific Research Institute of Neurosciences and Medicine, Novosibirsk, Russia; Institute of Cytology and Genetics of the Siberian Branch of the Russian Academy of Sciences, Novosibirsk, Russia

**Keywords:** Clstn2-KO mice, social behavior, brain, ventral tegmental area, dopaminergic neurons, мыши Clstn2-KO, социальное вознаграждение, мозг, вентральный тегментум, дофаминергические нейроны

## Abstract

The incidence of autistic spectrum disorders (ASD) constantly increases in the world. Studying the mechanisms underlying ASD as well as searching for new therapeutic targets are crucial tasks. Many researchers agree that autism is a neurodevelopmental disorder. Clstn2-KO mouse strain with a knockout of calsyntenin 2 gene (Clstn2) is model for investigating ASD. This study aims to evaluate the social-conditioned place preference as well as density of dopaminergic (DA) neurons in the ventral tegmental area (VTA), which belongs to the brain reward system, in the males of the Clstn2-KO strain using wild type C57BL/6J males as controls. Social-conditioned place preference test evaluates a reward-dependent component of social behavior. The results of this test revealed differences between the Clstn2-KO and the control males, as the former did not value socializing with the familiar partner, spending equal time in the isolation- and socializing-associated compartments. The Clstn2-KO group entered both compartments more frequently, but spent less time in the socializing-associated compartment compared to the controls. By contrast, the control males of the C57BL/6J strain spent more time in socializing-associated compartment and less time in the compartment that was associated with loneness. At the same time, an increased number of DA and possibly GABA neurons labeled with antibodies against the type 2 dopamine receptor as well as against tyrosine hydroxylase were detected in the VTA of the Clstn2-KO mice. Thus, a change in social-conditioned place preference in Clstn2-KO mice as well as a higher number of neurons expressing type 2 dopamine receptors and tyrosine hydroxylase in the VTA, the key structure of the mesolimbic dopaminergic pathway, were observed.

## Abstract (ru)

В мире наблюдается рост случаев расстройств аутистического спектра (РАС). Исследование механизмов и причин возникновения РАС, а также поиск мишеней для терапии этих расстройств являются актуальной задачей. Многие исследователи сходятся во мнении, что возникновение аутизма связано с нарушением развития нервной системы. Линия мышей Clstn2-KO, нокаутов по гену кальсинтенин-2, полученная на основе C57BL/6J, моделирует симптомы РАС. Данное исследование было направлено на изучение у самцов Clstn2-KO социального предпочтения места и плотности дофаминергических нейронов в вентральном тегментуме, который представляет собой часть системы вознаграждения головного мозга, в сравнении с контрольной линией мышей C57BL/6J дикого типа. Тест «социально обусловленное предпочтение места» отражает социальное вознаграждение. Результаты этого теста показали, что у самцов Clstn2-KO наблюдаются отклонения от контрольных мышей в социальном вознаграждении, так как они проводили одинаковое время в обоих отсеках установки, ассоциированных либо с изоляцией, либо с социализацией со знакомым партнером. При этом животные из группы Clstn2-KO заходили в обе части камеры зна- чительно чаще, но проводили меньше времени в социально-ассоциированном отсеке по сравнению с контрольной группой. Самцы контрольной линии C57BL/6J, напротив, проводили больше времени в отсеке, ассоциированном с социализацией, где было взаимодействие с сородичем, и меньше в отсеке, в котором ранее особь находилась в оди- ночестве. В вентральном тегментуме, отвечающем за процессы, связанные с вознаграждением, у мышей Clstn2- KO было обнаружено повышенное число дофаминергических нейронов и, возможно, ГАМК-ергических нейронов, меченных антителами против дофаминового рецептора второго типа и тирозингидроксилазы. На основании по- лученных результатов можно заключить, что у мышей Clstn2-KO имеет место изменение значимости социального вознаграждения, а также обнаружено повышенное число нейронов, экспрессирующих дофаминовые рецепторы второго типа и тирозингидроксилазу, в одной из важных структур мезолимбического дофаминергического пути – вентральном тегментуме, который является частью системы вознаграждения.

## Introduction

Autism Spectrum Disorders (ASD) in children are characterized by impaired social interaction, low interest in peers, and difficulties in maintaining social contacts (Autism Spectrum Disorder, 2013). Many researchers agree that the ASD are developmental disorders of the nervous system (Bourgeron, 2009; Buxbaum, 2009; Marshall, Mason, 2019; Sawicka et al., 2019; Girault, Piven, 2020; Yang, Shcheglovitov, 2020). An imbalance between excitation and inhibition processes in various brain structures is characteristic for ASD (Canitano, 2007), which is caused by abnormal interactions between neurons and by impaired synaptic plasticity (Zoghbi, 2003). Mutations in the adhesion proteins genes, which play a key role in intercellular connections, including interneuronal and neuroglial contacts, have been identified in a number of ASD studies (Bourgeron, 2009; Buxbaum, 2009). In particular, impaired synthesis of neurexins, neuroligins, contactins, and cadherins may be associated with the development of ASD in humans (Bourgeron, 2009; Buxbaum, 2009). Also, in the mouse strains modeling these disorders, the expression of genes responsible for the formation of these proteins may be impaired (Lipina et al., 2016; Zhang Q. et al., 2019).

Calcintenin-1, -2 and -3 (Clstn1, Clstn2 and Clstn3), belonging to the cadherin family, are synaptic adhesion proteins that are able to bind Ca2+ ions and regulate their intracellular concentration. Of particular interest is Clstn2, which is specifically expressed in inhibitory interneurons (Hintsch et al., 2002) and is associated with verbal memory in adolescents (Jacobsen et al., 2009), as well as with semantic and cognitive characteristics in the elderly (Laukka et al., 2013). Moreover, genetic analysis of gene copy number variation in autistic patients revealed a deletion of the 2nd intron of the Clstn2 gene (AlAyadhi et al., 2016). According to The Human Protein Atlas (https://www.proteinatlas.org/), Clstn2 in mice is expressed in the hippocampus and some other brain structures, including the midbrain. To study the function of this protein, a Clstn2 knockout (Clstn2-KO) mouse strain based on C57BL/6J was established (Lipina et al., 2016). As we have shown earlier, the absence of Clstn2 in mice causes a selective deficit of inhibitory interneurons in the prefrontal cortex and hippocampus (Lipina et al., 2016). This is accompanied by the manifestation of ASD-like conditions, including stereotypy, insufficient social motivation, abnormal ultrasonic vocalization (Ranneva et al., 2017; Klenova et al., 2021), as well as morphological changes in synapses (Ranneva et al., 2020).

Previously, structural and functional disorders in the mesolimbic dopaminergic pathway, which includes the ventral tegmental area (VTA) and nucleus accumbens, were found in children with ASD, and these changes in the reward system were demonstrated to be associated with underdevelopment of social skills (Supekar et al., 2018). Studies demonstrate that synaptic proteins associated with the development of ASD (Huguet et al., 2016) play an important role in the functioning of the mesolimbic pathway of the dopaminergic (DS) and GABAergic brain systems (Hart et al., 2012; Karayannis et al., 2014), one of the key midbrain components of which is the VTA (Lammel et al., 2008; Morales, Margolis, 2017). The VTA is a key structure of the reward brain system (Sesack, Grace, 2010) and regulates behavioral response to reward/ punishment, including social reinforcement (Gunaydin, Deisseroth, 2014; Saunders et al., 2015). The VTA contains the bodies of dopaminergic (DA) neurons, as well as the glutamatergic and GABAergic neurons (Saunders et al., 2015). Terminals of DA neurons of the dopamine mesolimbic pathway are characterized by co-transmission, i. e. the ability to release various neurotransmitters, in particular, dopamine, glutamate, and GABA (Root et al., 2014; Zhang S. et al., 2015; Berrios et al., 2016).

One of the theories of autism is based on the notion that social motivation is reduced in autistic persons due to the alterations in the brain reward system (Kohls et al., 2012). Although the development of subcortical neuronal mechanisms of the brain is critical within the first months of life, the brain structures involved in the reward processes, that is, in the formation and correction of behavior through positive reactions to various stimuli, are functioning during the lifespan (Kohls et al., 2012, 2014). The imbalance between social and non-social motivation is the peculiar characteristic of the reward system in autistic persons (Kohls et al., 2014). This theory assumes that the reward system in ASD subjects is hyperactive in response to interests unrelated to socialization, while disruption of social behavior associates underactivity of the brain reward system in response to socially significant stimuli (Kohls et al., 2012, 2014).

The neurobiological reward system includes DA neurons of the VTA, which have projections mainly to the nucleus accumbens and to the prefrontal cortex, and regulates social motivation (Saunders et al., 2015). It was demonstrated that DA neurons of the reward system increase their activity during the interaction of a mouse with a relative (Solie et al., 2022). A characteristic feature of DA neurons is that they release dopamine as a neurotransmitter and also contain the enzyme tyrosine hydroxylase (TH), which is necessary for its synthesis (Morales, Margolis, 2017). The study of Lammel et al. (2008) considers two types of DA neurons in the VTA (Lammel et al., 2008). Type 1 DA neurons express TH and a dopamine receptor type 2 (D2R), and their terminals end up within the shell of the nucleus accumbens and in the dorsolateral striatum. Type 2 DA neurons express TH, but not D2R, and their endings spread to the prefrontal cortex, the core and the medial zone of the shell of the nucleus accumbens, as well as the basolateral parts of the amygdala nuclei. The D2R, which can be expressed not only on DA but also on GABAergic neurons (Lammel et al., 2008; Margolis et al., 2012; Morales, Margolis, 2017), is associated with addictive behavior in which the brain reward system, the VTA in particular, is involved (Bello et al., 2011).

Mice express social behavior in a variety of contexts, including interactions with peers of the same and the opposite sex, it is also involved in early play behavior and in mother-offspring interactions (Chen, Hong, 2018). The socialconditioned place preference test evaluates social reward in young and adult mice when a certain context is associated with positive social interaction with a familiar partner (Panksepp, Lahvis, 2007; Lipina et al., 2013; Lan et al., 2019). Based on this, we hypothesized that ASD-like social behavior may be associated with impaired functioning of one or more elements of the mesolimbic dopaminergic pathway, which plays an important role in the regulation of social preference (Gunaydin, Deisseroth, 2014).

The aim of this work was to study social reward in Clstn2 knockout mice (Clstn2-KO), as well as to study the density of neurons containing D2R and TH in the VTA.

## Materials and methods

Experimental animals. Seven Clstn2-KO males, and five wild-type (C57BL/6J) males at the age of three months were used in this study. Animals were kept in the same-sex groups of 3–5 individuals in 36 × 25 × 14 cm (length × width × height) cages, in a conventional vivarium at the Institute of Neurosciences and Medicine (Novosibirsk) with sawdust bedding; 12D:12L cycle, at 20–22 °C, with free access to dry granulated food for laboratory rodents and to purified water. All studies were done in accordance with the European Convention for the Protection of Vertebrate Animals used for Experimental and Other Scientific Purposes (ETS No. 123).

Social-conditioned place preference test. The study was carried out as described previously (Panksepp, Lahvis, 2007; Lipina et al., 2013; Lan et al., 2019), with minor modifications. Briefly, on the eve of the experiment, the animals were kept individually for 24 hours. The experimental chamber consisted of three compartments. Two outer compartments (between which there was a third – an intermediate compartment) were separated by removable partitions. The floor, made of polypropylene, was of a different texture in the two outer compartments: rough and smooth. Before testing, the mice were adapted to the experimental cage and the selection of the floor texture by the tested mouse was evaluated to exclude the possibility of the preference for one or another surface non-related with social interaction (session “adaptation”). The assessment was carried out visually using a stopwatch: the time spent (in seconds) in each of the compartments during 20 minutes. After adaptation, the test animals were housed in separate cages for 24 hours. Thereafter, the main experiment started.

The compartment with a rough surface was associated with social interaction, as the studied mouse was there in contact with its familiar relative of the same sex, age, and genotype, while the compartment with a smooth surface was associated with isolation, as the mouse was alone there. The procedure for establishing an “association” of the surface type with the compartment context took three days. On the first day of the experiment, the tested mouse was placed for 20 minutes in a compartment with a rough floor for socialization with a familiar partner. Three hours later, the animal was transferred to a compartment with a smooth floor, where it was left alone for 20 minutes. On the second day, the test mouse was first placed in the smooth surface compartment, where it was alone for 20 minutes, and after three hours, it was placed in the rough surface compartment with a partner for 20 minutes. On the third day of the experiment, the conditions were repeated as described for the first day. It is important to note that the familiar partner during the 20-minute socialization remained the same for each experimental animal during the three days of social reward formation. After each 20-minute session, surfaces were cleaned up with 70 % alcohol to remove odors and the surfaces were thoroughly dried. On the fourth day, the mice explored the set for 15 minutes (basic behavior session). On the fifth day of the experiment (“social reward test”), each mouse was placed in the central compartment of an empty setup, the partitions were removed to allow free movement, and the time spent in the compartments with a smooth and rough floor texture was recorded for 20 minutes. The evaluation was carried out visually using a stopwatch. The criterion for the presence of a mouse in a particular compartment was the presence of the entire body of the animal (all four paws) in the compartment, either with a rough or smooth floor covering.

Intracardiac perfusion. All animals used in the behavioral experiment were perfused the day after its completion through the circulatory system to fix the brain. Mice were anesthetized by intramuscular injection of 75 μL (per 10 g of weight) medetomidine hydrochloride (Meditin, 1 mg/ml; APISAN, Russia) and 60 μL (per 10 g of weight) zoletil (Virbac, France). Thereafter, mice were injected through the circulatory system with 30–50 mL of phosphate-buffered saline (PBS), and then 10 % formalin solution based on PBS. After that, the brain was removed and placed in a 30 % sucrose solution in PBS at +4 °C for dehydration and further fixation for the next 3–4 weeks until the fixed material sank to the bottom of the flask. The fixed brain samples were frozen using Tissue-Tek O.C.T. (Sakura Finetek, USA) and stored at –70 °C.

Preparation of frozen brain slices. Three animals were randomly chosen for each group for the histological analysis. Frozen brain sections from each of the animals were made at a distance of –2.92 to –3.28 mm from the bregma, which corresponds to the area of the VTA. Sections 10 μm thick were obtained on an HM550 OP Cryotome (Thermo Fisher Scientific, USA) at –25 °C and placed on Superfrost Plus, Menzel-Glaser glass slides (Thermo Fisher Scientific).

Immunohistochemical staining. Sample staining was performed according to the manufacturer’s protocols with minor modifications. Briefly, after washing and exposure to Protein Block ab64226 (Abcam, UK), 50 μL of the corresponding antibody was added and left in a humid dark chamber overnight at +4 °C. The concentration of antibodies was: 1:400, 1:800 – anti-D2R-AF647 sc-5303 (Santa Cruz Biotechnology, USA) and anti-TH-AF488 MAB318-AF488 (Merck, Germany), respectively. Thereafter, the samples were washed in PBS-Tween, excess liquid was removed and placed in ProLong, Glass Antifade Mountant, Thermo P36982 (Thermo Fisher Scientific).

Analysis of the density of neurons. Images were obtained using a confocal laser scanning microscope LSM 780 (Carl Zeiss, Germany) equipped with a Plan-Apochromat 20x/0.8 M27 objective (Carl Zeiss) at the research facilities of the Center for Collective Use of Microscopic Analysis of Biological Objects of the Siberian Branch of the Russian Academy of Sciences (https://ckp.icgen.ru/ckpmabo/) to estimate the density of antibodies labeled neurons. The number of cells was counted manually: without the use of special programs for counting, in at least three sections per animal, in a field of view of 10 000 μm2 (one field of view per section). Since the VTA is a heterogeneous structure (Sanchez-Catalan et al., 2014), we took sections throughout the entire area, which correspond to a certain distance from the bregma, i. e., the rostral part of the VTA –2.92 mm, the central part –3.16 mm and caudal part –3.28 mm according to the atlas (Paxinos, Franklin, 2001). The ImageJ program was used to restrict the field of view (10 000 μm2). The average number of cells from three sections for each animal and the average volume density (mm3) were calculated.

Statistical analysis. The analysis of the results was carried out using the STATISTICA v. 12.0 (StatSoft, Inc., USA) software package. All data were tested for normality using the Shapiro–Wilk W-test. Data on the behavioral parameters are presented as mean ± standard error of the mean (M ± SEM). Comparison between groups was performed using Student’s t-test. Data on neuron density are presented as a median with the first and third quartiles – Me [Q1;Q3]. The density of labeled neurons between groups was compared using the Mann–Whitney U-test. The significance level was taken at p <0.05.

## Results

The preliminary testing of the control (C57BL/6J) and Clstn2- KO mice before the start of the main experiment did not reveal significant differences on the time spent in the compartments with smooth (499.8 ± 43.6 and 490.5 ± 37.0 sec, respectively) and rough (550.7 ± 17.8 and 472.8 ± 28.3 sec, respectively) floor; thus the preference for a certain compartment by mice of both studied groups was excluded. The results of the main test are presented in the Table. Mice of the control group spent more time ( p < 0.05) in the socially associated compartment, where there was interaction with the conspecifics, compared with the compartment in which the individual was previously alone. Meanwhile, Clstn2-KO mice spent the same amount of time in both compartments. At the same time, animals from the Clstn2-KO group entered both parts of the chamber much more often ( p < 0.001), but spent less time ( p < 0.05) in the socially associated compartment compared to mice of the control group.

**Table 1.**
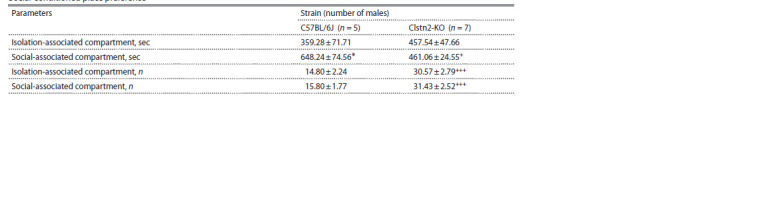
Social-conditioned place preference *p < 0.05 as compared with time in isolation-associated compartment; +p < 0.05 as compared with C57BL/6J; +++p < 0.001 as compared with C57BL/6J.

**Fig. 1.**
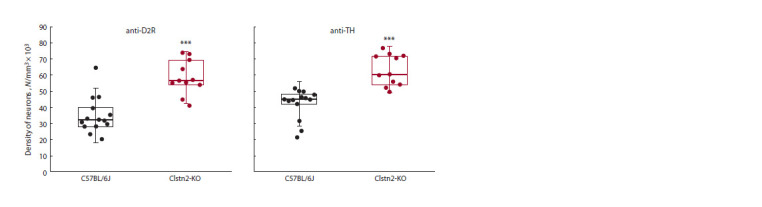
The density of neurons labeled with anti-D2R, and anti-TH in the ventral tegmental area. N – number of neurons in the field of interest. ● – density of neurons obtained per each slice. The upper and the lower bounds of the boxes correspond to the first and the third quartiles, respectively; bold horizontal line – median; vertical lines – standard deviation. ***p < 0.001 as compared with C57BL/6J.

**Fig. 2.**
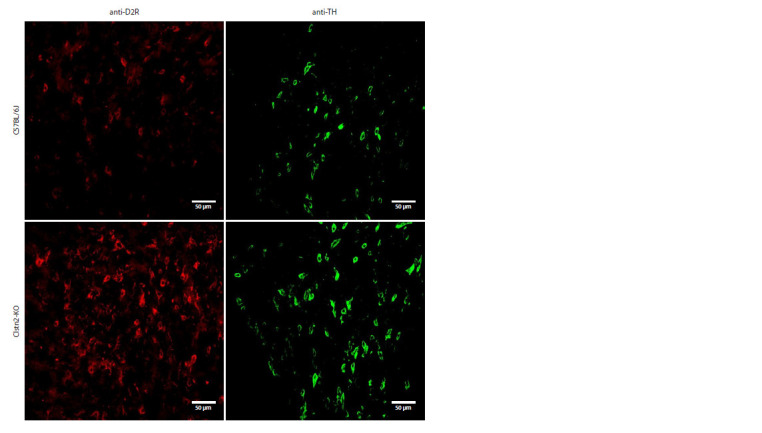
The density of neurons labeled with antibodies against the second dopamine receptor (anti-D2R-AF647) and tyrosine hydroxylase (anti-TH-AF488) in males of C57BL/6J and Clstn2-KO strains in the ventral tegmental area.

Data on the density of VTA neurons labeled with D2R and TH antibodies are presented in Figures 1 and 2. Statistical analysis revealed a higher ( p < 0.001) density of neurons labeled with anti-D2R and anti-TH in Clstn2-KO knockout mice in the studied area as compared to controls.

## Discussion

Previously, the social-conditioned place preference test was already used on mice of different strains (C57BL, DBA, BALB, Disc1-Q31L) (Panksepp, Lahvis, 2007; Lipina et al., 2013; Lan et al., 2019). It was shown that normally the animals spend more time in the compartment where they had previously contacted conspecifics; these findings are consistent with the notion that socially conditioned place preference reflects social rewards (Panksepp, Lahvis, 2007). In our study, we examined social place preference as well as the density of anti-D2R and anti-TH antibody-labeled neurons in the VTA of Clstn2-KO males and wild-type control (C57BL/6J) mice. In the social-conditioned place preference test, Clstn2-KO mice entered both compartments significantly more often, which is apparently due to their higher level of locomotor activity compared to the controls, which is consistent with the hyperactivity of these animals described in an earlier work (Lipina et al., 2016). It is possible that Clstn2-KO mice, due to their hyperactivity, were unable to form a reward caused by daily socialization with a familiar partner, and as a result, were unable to express their preference for the “social” compartment. The observed impairment of social place preference in Clstn2- KO mice is in good agreement with the previously reported impairment of social behavior for these mice (Ranneva et al., 2017; Klenova et al., 2021).

In our work, we focused on the study of neurons expressing D2R and TH in the VTA. In both Clstn2-KO and C57BL/6J mice, the number of neurons labeled with anti-TH antibodies was slightly higher than the number of neurons labeled against D2R. This is apparently due to the fact that not only the DA neurons in which TH is found but also GABAergic neurons of the VTA express D2R (Lammel et al., 2008; Morales, Margolis, 2017). Meanwhile, we found more neurons with both D2R and TH in the VTA of Clstn2-KO mice compared to controls.

It was found that Clstn2-KO mice have more neurons containing D2R, as well as TH in the VTA compared to C57BL mice. Our data, as well as the results obtained on other strains of mice modeling ASD (Squillace et al., 2014; Bariselli et al., 2016, 2018; Chao et al., 2020; Tassan Mazzocco et al., 2021), indicate changes in the mesolimbic dopaminergic pathway, which also plays an important role in human ASD (Supekar et al., 2018). In particular, in the work on mice of the BTBR strain, despite the fact that they did not reveal functional changes in D1R in the striatum, a sharp decrease in D2R functions was observed upon activation of DA neurons (Squillace et al., 2014). Also, in Shank3 and Nlgn3-KO mice, a decrease in the activity of DA neurons in the VTA was revealed, which caused a behavioral deficit, including alterations of social preferences compared to C57BL controls (Bariselli et al., 2016, 2018). In another study, two strains of mice modeling different forms of ASD were studied: BTBR and Fmr1-KO (Chao et al., 2020). A general decrease in tyrosine hydroxylase expression was found in the substantia nigra, VTA and striatum and in BTBR mice compared to C57BL mice, but not in the Fmr1-KO strain (Chao et al., 2020). In a study of TKO mice, which is another model of autism, no changes were found in the VTA DA neurons (Tassan Mazzocco et al., 2021).

Thus, ASD is often, but not always, associated with disturbances in the DS in the VTA. A rather unexpected result is that in Clstn2-KO mice the DS in the VTA is changed, but in the direction of an increase in the number of neurons containing D2R and TH. The previously described hyperactivity of Clstn2-KO mice (Lipina et al., 2016), which was corroborated in the current work by the increased frequency of entering of the compartments in the social-conditioned place preference test, may be associated with an increased density of neurons expressing D2R. It has been shown in the hyperactive Coloboma mice, that knockout of the D2R dopamine receptor gene resulted in a decrease in locomotor activity compared to controls (Fan et al., 2010). Based on this, one may assume that the increase in neurons with D2R in the VTA of Clstn2-KO mice reported herein may be associated with an increased locomotor activity of these animals. It is also interesting to note that human studies have shown that nucleotide polymorphism in the D2R gene can be considered as a potential risk factor for the development of not only ASD, but also attention deficit hyperactivity disorder (Mariggio et al., 2021).

It was previously shown that male Disc1-Q31L mice with depression-like behavior, which were studied in the socialconditioned place preference test, unlike Clstn2-KO mice, preferred the compartment associated with isolation (Lipina et al., 2013). It can be assumed that this test adequately assesses the alterations of social behavior different models of mental disorders. Indeed, a depressive-like state caused by a deficiency of monoamines, including DA, is characterized by a complete avoidance of social contacts, which was demonstrated for the Disc1-Q31L strain (Lipina et al., 2013). However, in our study on Clstn2-KO mice, which are a model of ASD, results of this test were different. Nevertheless, we cannot completely exclude the effect of impaired spatial longterm memory observed in the Morris test in Clstn2-KO mice (Lipina et al., 2016) on social preference, which needs to be considered in future studies.

The data obtained may indicate a decrease in motivation for interacting with conspecifics in mice with a knockout for the Clstn2 gene, as the mice of this strain have not demonstrated preferences to social-associated compartment. Also, changes were found in the VTA, which plays an important role in social preference (Gunaydin, Deisseroth, 2014); in this brain structure, an increased number of neurons expressing D2R and TH was found in Clstn2-KO mice. Thus, it can be assumed that the Clstn2 gene plays a certain role in dopamine-dependent processes of reward and motor activity, which may be associated with changes in the density of DA neurons in the VTA.

## Conclusion

The results of this study suggest that Clstn2 knockout mice, which can be considered as a model for studying autism spectrum disorders, demonstrate a change in the perception of social reward and an increased number of neurons expressing dopamine type 2 receptors and tyrosine hydroxylase in one of the important structures of the mesolimbic dopaminergic pathway – the ventral tegmental area, which is part of the reward system.

## Conflict of interest

The authors declare no conflict of interest.
